# The Underlying Mechanisms of Psychological Resilience on Emotional Experience: Attention-Bias or Emotion Disengagement

**DOI:** 10.3389/fpsyg.2020.01993

**Published:** 2020-09-17

**Authors:** Feng Yi, Xiaofang Li, Xiaolei Song, Lei Zhu

**Affiliations:** ^1^School of Psychology, Key Laboratory for Behavior and Cognitive Neuroscience of Shaanxi Province, Shaanxi Normal University, Xi’an, China; ^2^People’s Government of Anhua Town, Lanzhou, China

**Keywords:** psychological resilience, emotional experience, attention-bias, emotion disengage, input–output model

## Abstract

Psychological resilience is consisted of social resources and protective factors for individuals against negative effects, and can influence the process of meta-cognition of individuals in response to emotion feelings. However, individuals with high or low resilience may produce various emotional experiences when facing the same events. According to an emotional input–output model, the different impacts of resilience on emotional experience may be caused during the process of receiving or disengaging stages. In order to address this problem, three experiments were conducted in the present study. The Experiment 1 was designed to explore whether the positive and negative emotions were associated with higher or lower levels of resilience. The aims of Experiments 2 and 3 were to test at which stages the different emotional experiences were caused by high or low resilience of individuals. The results showed that individuals with low resilience were more likely to feel more negative and less positive emotions, and resilience was significantly negatively associated with anxiety or depression. However, there was no difference in the stage of receiving emotional information between high and low resilient individuals, but differ on their ability of disengagement from emotional information, the individuals with high resilience disengaged from both positive and negative emotional information much faster. These findings were discussed in the context of different theories about the relationship between resilience and emotional experience.

## Introduction

In our daily life, there are various emotional information abound with us. Positive emotion is important for individuals’ psychological health, referring to individuals’ pleasant experiences and feelings when their needs were met (Wang et al., 2010). The Broaden-building theory of positive emotion by Fredrickson (2001, 2015) suggested that positive emotions such as happiness, interest, satisfaction, and love extended individuals’ mind and action sequence. Happiness stimulated their desire for playing and exploration, interest met their desire to light and integrate, and love make their desires circulating repeatedly in a safe and intimate relationship. All above can help individuals get into society more smoothly, thus establish personal resources, including the physical and intellectual ones, which are salutary for individuals to cope with the challenges from the world (Wang et al., 2010). A number of research has showed that positive emotion possesses several other benefits: (a) it increases trust and agreement with acquaintances or close people (Dunn and Schweitzer, 2005; Fredrickson et al., 2008); (b) it is conducive to the development of friendship and marital satisfaction (Harker and Keltner, 2001); (c) it promots happiness (Fredrickson and Joiner, 2002), and offsets the undesirable effects of negative emotion to a certain degree (Fredrickson and Levenson, 1998); and (d) it can promote individuals to adapt to their environments and cope with adversity and challenge properly (Folkman and Moskowitz, 2000, 2004), which is helpful for individuals to extricate from their major stress events (Tugade and Fredrickson, 2004), and upgrade their resilience (Cohn et al., 2009) and their physical and mental healths (Koivumaahonkanen et al., 2004; Diener et al., 2006; Doyle et al., 2006). However, individuals differ greatly on perceiving, receiving, and processing of their emotion information (Ding et al., 2007). Although facing the same events, some people indeed feel more pleasant. Thus, a growing number of researchers try to understand why some are able to experience a higher level of positive emotion and the well-adjusted in their lives. Finally, It was found that emotional experience was influenced by the level of psychological resilience, and there were many factors affect individuals’ experience of emotional material, such as anxiety and depression state (Joormann and Gotlib, 2007) and psychological resilience level (Tugade and Fredrickson, 2004).

Resilience is usually defined as a human trait, referring to one’s ability to resist, recover, and bounce back from the negative effects of stress and adversity (Lazarus, 1993; Leipold and Greve, 2009; Chmitorza et al., 2018), and even develop much better than before. Recent research has shown that resilience consists of various factors that can enhance individuals’ personal resources, protect them from stress and negative evaluation (Kalisch et al., 2015), which has an effect on the meta-cognition of response to emotions (Fletcher and Sarkar, 2013), and involve their ability to experience emotions mapping the situation (Reynaud et al., 2013). In addition, some researchers point out that there is a distinct connection between resilience and emotion regulation (Kay, 2016). Hence, we reasonably consider that psychological resilience plays an important role in emotional experience.

As expected, a number of studies about emotional state consistentlyshowed that high resilient individuals reported a higher level of positive emotion than low resilient ones (Fredrickson, 2003; Tugade and Fredrickson, 2004; Wang and Wang, 2013), and they can use fun, wit, and humor to develop positive emotions for themselves to overcome difficulties and adversities in their lives (Tugade and Fredrickson, 2004). A study of Eaton et al. (2007) showed that positive emotions were a core factor of extraversion and resilience. But the results of these research about negative emotion among individuals with different resilients are conflicting. Tugade and Fredrickson (2004) found that there was no significant differences in the negative emotion level between high and low resilient individuals, while Bonanno et al. (2007) discovered that higher resilience was significantly correlated with low depression level, and comparing to individuals with low resilience, adverse childhood experiences had less influence on emotional dysregulation of the individuals who have a higher level of resilience (Poole et al., 2017). Ong et al. (2006) conducted an interview study and even found that high resilient individuals in stress could feel positive emotion and negative emotion, but low resilient individuals can only feel negative emotion after disasters. Considering these conflicting results, more studies should be done to clarify the influence of resilience on negative emotional experience.

Emotional experience is a complex process, including selective attention, that can be encoded to emotional information and ultimately disengaged from the current mood (Gross, 1998). Based on the process of emotion occurrence, Gross (1998) proposed an emotion input–output model, which suggested that individuals’ emotion could be regulated either at the input or disengaging stages of the emotional information. If the emotion is regulated in the input stage (cause regulation), which would directly adjust the evaluation experience process that produces emotion, such as enhance evaluation (emphasis) or weaken evaluation (neglect). On the contrary, if the regulation occurs in the disengagement stage, evaluation of emotional valence would be determined by emotional response. When individuals has made corresponding responses to emotional information, they would separate themselves from the mood, and the evaluation of this kind of emotion was gradually weakened. While if someone couldn’t do response timely, or spend more time disengaging from the emotion, they would have more intense experience.

Gross and Thompson (2007) suggested that if an individual did not focus on negative stimuli, he or she would assess the environment as low threatened, and perceive less negative emotions. Joormann and Gotlib (2007) used negative emotion priming tasks to measure individuals’ attention bias of emotional words and found that individuals in anxiety state had an attention bias toward the negative induction words. They also used dot-probe tasks and found that high risk depressed children showed an attention bias toward the negative emotion faces, meanwhile low depressed children showed an attention bias toward the positive emotion faces (Joormann and Gotlib, 2007). A study using eye tracking showed that the optimistic individuals noticed more positive information and the pessimistic ones noticed more negative information (Isaacowitz, 2005). Song (2013) used dot probe tasks and showed that in the initial stage of attention, all the high and low level resilient groups showed attention bias toward the negative emotional information. There was no difference between the two groups; but in the later stage of attention, there was no significant attention bias in the high resilient group, while the low resilient group still had attention bias toward the negative emotional information. Hence we consider it is necessary to explore the effect of different psychological resilience on emotional experience from input and output stages separately. The current questions are that whether there is a difference among different resilient people in perceiving or disengaging emotional information, and if there is, at which stage (perceiving/disengaging stages) this difference occurs. If individuals could not focus on the positive information, or disengage themselves from the negative emotion stimuli effectively, they would have a more dangerous cognitive evaluation to the stressors, and thus experience more persistent negative emotions, and feel more difficult to maintain their mental health.

Based on the previous research and the input–output model of emotion, we planned to explore the influence of resilience on emotional experience from two stages of emotion information. It was hypothesized that high resilient participants would have an attention bias to positive emotional information, and disengage themselves from negative emotional information more quickly than the low resilient ones did. It should be noted that anxiety and depression may have an effect on experiences of emotional material, and thus, we were also interested in examining the relationships between their states and resilience. Hence, three experiments were conducted in present study. The aim of the Experiment 1 was to test whether there were differences between high and low resilient individuals in experiencing the emotional information; the Experiments 2 and 3 were designed to examine the styles of two groups in attention and disengagement of emotional pictures, and explore possible reasons of different experiences between high and low resilient groups. All Experiments measured the anxiety and depression level of each subject.

## Experiment 1

The aim of Experiment 1 was to examine whether there were differences between high and low resilient people in perceiving emotional information (positive, negative, and neutral) by a 9-point Likert scale.

### Materials and Methods

#### Participants

Sixty Chinese college undergraduates (30 in each of the high and low resilient groups) were recruited to take part for credits toward a course requirement and a bit of money for their participation. All participants had normal or corrected-to-normal vision, with no history of attention deficit, and all signed the written consent form. In order to determine the sample size, the G^∗^power 3.1.9.2 software was employed, which was designed as a flexible statistical power analysis program for statistical tests commonly used in social and behavioral research (Faul et al., 2007). Considering the test family (i.e., t tests), statistical test (i.e., Means: Difference between two independent means), the type of power analysis (i.e., the priori), the alpha-level for the previous and current study (i.e., 0.05), a desired level of statistical power of 0.8, the effect size (i.e., 0.8, identified as the large level in the software) as well as allocation ratio N2/N1 (1) revealed a sample size of 42 participants in total. The current study also calculate the correlation. When the statistical test was changed to the option of correlation, and other parameters were same, only 21 participants were needed. However, we planned to test 60 participants to make sure that the effect size was in a high level. The same method were used to select the sample size in Experiments 2 and 3, indicating that there were 10 participants at least.

The participants were initially screened using ego-resiliency scale (ER89; Yu and Zhang, 2007), Self-Rating Anxiety Scale (SAS; Zung, 1971), Self-rating Depression Scale (SDS; Zung, 1965), which was measured in a group of 110 undergraduates. Except for students with high anxiety and high depressive symptoms, according to the usual standards of psychometrics (Zheng et al., 1998, p. 27), students who scored within the top 27 and low 27% on Ego-resiliency scale were invited to the lab as high and low resilient group to complete the affective rating procedures. High (*M* = 48.27, SD = 2.67, 28 male) and low (*M* = 32.73, SD = 3.72, 28 male) group had different scores on ego-resiliency scale, *t*(58) = −18.96, *p* < 0.01, *d* = 3.278.

#### Materials

##### Ego-Resiliency Scale

The Chinese version (Yu and Zhang, 2007) of Block and Kremen’s ER89 (Block and Kremen, 1996) was used to assess trait variation in psychological resilience. Participants were asked to indicate the degree to which they agreed with 14 statements (e.g., “I quickly get over and recover from being startled”) on a Likert scale ranging from 1 (*does not apply at all*) to 4 (*applies very strongly*). The total score can be calculated by adding all items. The higher the total scores, the higher the psychological toughness. The Chinese version of ER89 has been shown to have high construct validity (Yu and Zhang, 2007). In the current sample, the Cronbach’s alpha was 0.78.

##### Self-Rating Anxiety Scale

Zung’s SAS (Zung, 1971) was used to assess individuals’ symptoms of anxiety, including 20 items that can be answered from 1 (*does not apply at all*) to 4 (*applies very strongly*). Individuals with a total score higher than 40 were considered to have clinically elevated levels of anxiety. The Chinese version of SAS had shown high construct validity (Wang et al., 2005). In this study, the Cronbach’s alpha was 0.73.

##### Self-Rating Depression Scale

Zung’s SDS (Zung, 1965) was used to assess individuals’ symptoms of depression including 20 items that can be answered from 1 (*does not apply at all*) to 4 (*applies very strongly*). Individuals with a total score higher than 40 were considered to have elevated levels of depression. The SDS has been shown to have good construct validity (Yu et al., 2005). In this study, the Cronbach’s alpha was 0.74.

##### Emotional Feelings

From the existing studies, there are three major methods to measure emotional experience, including instrument measurement, over behavior and self-report scales, respectively (Bai and Guo, 2010). This study employed emotional degree scales to measure emotional experience, where participants were asked to rate their emotional feelings about the pictures presented in the center of the screen on a nine-point scale ranging from 1 (*very unpleasant*) to 9 (*pleasant very much*). In total, 42 pictures (14 for each of positive, negative, and neutral) were chosen from the Chinese Affective Picture System (CAPS). The valence and arousal of pictures can be seen in Table 1. Arousal of positive picture was not different from negative picture, *t*(26) = 1.05, *p* > 0.05. The valence of positive picture was higher than that of neutral picture [*t*(26) = 26.94, *p* < 0.001, *d* = 8.08], and negative picture’s valence was lower [*t*(26) = −27.28, *p* < 0.001, *d* = 4.54].

**TABLE 1:** Valence and arousal of positive pictures (*M* ± SD).

	*n*	Valence	Arousal
Positive	14	7.45 ± 0.44	6.54 ± 0.36
Negative	14	2.72 ± 0.58	6.59 ± 0.34
Neutral	14	4.69 ± 0.20	2.42 ± 0.43

#### Materials

The materials were rendered by 19 inches screen desktop, with a resolution of 1024 × 768 pixels. Stimulus presentation, response recording and data collection were controlled by E-prime 2.0.

#### Procedure

A week before the lab experiment, participants completed a self-reported questionnaire including the ER89, SAS, and SDS scales. According to the scores of SAS and SDS, those with clinically elevated levels of anxiety and depression were excluded, then the high and the low resilient groups were invited to complete the affective rating procedures after a week.

**FIGURE 1:**
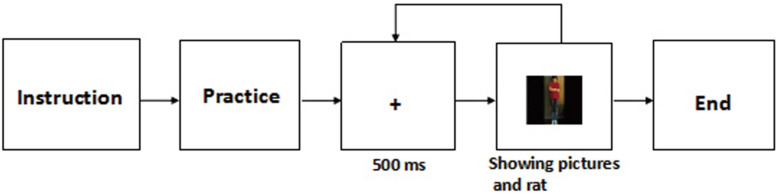
The procedure of emotional feelings rating.

In the emotional feeling rating task, Participants were tested individually and sat approximately 60 cm from the computer screen. They were asked to press the numbers on the keyboard to rate their valence feelings about the pictures. The task began with five practical trials, and followed by 60 experimental trials in which the three kinds of pictures were equally presented in a random order. Stimuli appeared until participants pressed the keyboard (1–9: 1-very unpleasant; 9-very pleasant) (see Figure 1). The background was white and words were black. All pictures were 9 cm × 10 cm (Guo and Lv, 2014).

### Results

The scores of all participants were within the normal range of the SAS and SDS scales. The high and low resilient participants were not significantly different in symptoms of anxiety, *t*(58) = 0.58, *p* = 0.57, *d* = 0.15, and depression *t*(58) = 1.99, *p* = 0.05, *d* = 0.52.

#### Correlation Analysis

Correlation analysis showed that resilience was significantly negative correlated with anxiety (*r* = −0.63, *p* < 0.01) and depression (*r* = −0.70, *p* < 0.01), and was significantly positive correlated with the rating of neutral pictures (*r* = 0.36, *p* < 0.01) and positive pictures (*r* = 0.31, *p* < 0.05); Meanwhile, anxiety was significantly positive correlated with depression (*r* = 0.69, *P* < 0.01); the rating of neutral pictures was significantly positive correlated with the rating of positive pictures (*r* = 0.49, *p* < 0.01) and the rating of negative pictures (*r* = 0.29, *p* < 0.05). The other variables were not correlated with each other (see Table 2).

**TABLE 2:** Descriptive statistics and correlations of variables.

Variables	*M*	SD	1	2	3	4	5	6
1. Psychology resilience	38.45	7.90	1					
2. Anxiety	38.77	6.56	−0.63**	1				
3. Depression	41.33	9.14	−0.70**	0.69**	1			
4. Rating of neutral picture	4.42	1.06	0.36**	–0.11	–0.25	1		
5. Ratingof positivepicture	7.82	0.98	0.31*	–0.24	–0.25	0.49**	1	
6. Rating of negative picture	1.46	0.46	0.29*	0.13	–0.12	0.29*	0.03	1

*p < 0.05. Correlation is significant at the 0.05 level, **p < 0.01is significant at the 0.05 level.

#### Regression

**FIGURE 2:**
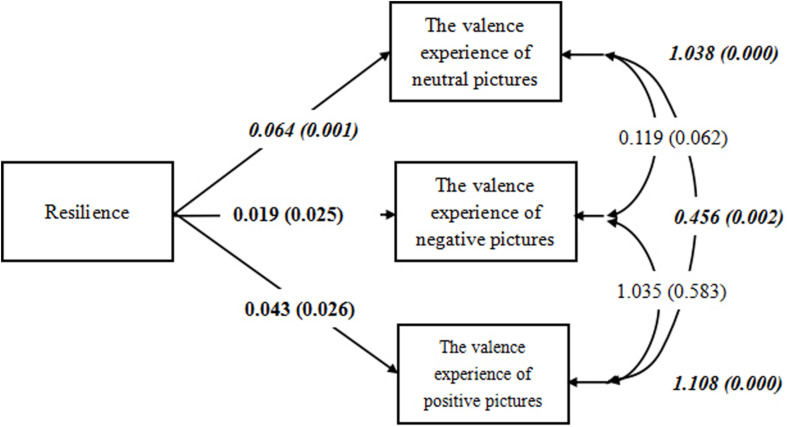
Complete MSEM model of Experiment 1. All results are standardized using the M-Plus SDTXY procedure. Values in bold are significantly different from zero at *p <* 0.05; values in bold and italics are significantly different from zero at *p* < 0.01.

To test the casual relationships between resilience and emotional experience, Mplus 7.3 was used, which allowed to test multiple effects simultaneously (Milyavskaya et al., 2015). The test result of the model was illustrated in Figure 2 (along with estimates of all parameters). When the score of resilience was employed as independent variable and the rating of three kinds of pictures were regarded as dependent variables. The results showed that resilience was significantly contributed to emotion feeling of three kinds of pictures (neutral: *p* = 0.001; positive: *p* = 0.026; negative: *p* = 0.025).

All results are standardized using the M-Plus SDTXY procedure. Values in bold are significantly different from zero at *p <* 0.05; values in bold and italics are significantly different from zero at *p* < 0.01.

#### Emotion Experiences of Different Resilient Groups

The results of independent samples *t*-test showed that the high and low resilient groups were significantly different in the neutral, positive, and negative emotional valences, with the high resilient group showing higher rating of three types of emotional pictures than the low group (all *p*s < 0.05) (see Table 3).

**TABLE 3:** Emotion experience of different resilient groups.

	Low resilient		High resilient				
	*M*	SD	*M*	SD	*t*	*p*	*d*
Neutral	3.90	0.96	4.94	0.89	–4.33	0.000	1.12
Positive	7.47	1.08	8.17	0.73	–2.94	0.005	0.76
Negative	1.31	0.33	1.61	0.53	–2.64	0.011	0.68

### Discussion

In Experiment 1, it was found that, rating of the valence of three kinds of pictures differed from each other, showed that individuals’ experience of positive, negative, and neutral pictures were significantly different. In addition to this, it was also found that resilience has effects on emotional experience, the high and low resilient groups rated the three kinds of pictures differently, with the high resilient group showing higher rating of three types of emotional pictures than the low group. This was consistent with the results of prior researches (Fredrickson et al., 2003; Tugade and Fredrickson, 2004). High resilient individuals rated negative pictures more positively maybe because they simultaneously experienced negative emotion as well as positive emotions (Ong et al., 2006). To explore whether resilience affected the experience of emotional information in the input (receiving) or disengaging stage of emotional information, Experiments 2 and 3 were conducted further.

## Experiment 2

As mentioned above, emotion was moderated at the input stage, which might have influenced the process of emotional experience and evaluation, such as enhancing experience (attention) or diminishing experience (ignore) that involved the attention bias (Gross, 1998). In other words, too much attention bias to emotional pictures would possibly enhance the emotional experience for individuals. While the Dot-probe task is often used to measure the attention bias, and the measurement can be used as selective attention to emotional information (Bocanegra et al., 2012), so a dot-probe task in Experiment 2 was used to test whether there were any differences in the receiving stage of emotional information.

### Materials and Methods

#### Participants

Participants were selected in the same way as Experiment 1. There were 58 participants recruited in this study (*M* age = 19.11 years, SD = 0.95), 28 (27 male) in high resilient group, and 30 (25 male) in low resilient group, the total score of the high psychological resilience group (*M* = 44.48, SD = 3.10) was significantly higher than that in the low psychological resilience group (*M* = 31.63, SD = 4.13), *t*(57) = 13.49, *p* < 0.001, *d* = 3.57. All participants had normal or corrected-to-normal vision, no history of attention deficit, and all signed the written consent form and were paid for a small monetary reward for the experiment.

#### Materials

Ego-resiliency scale, the Cronbach’s alpha was 0.88. Self-Rating Anxiety Scale, the Cronbach’s alpha was 0.74. Self-rating Depression Scale, the Cronbach’s alpha was 0.73. Emotional pictures being used in the Dot-probe task were same as Experiment 1.

#### Procedure

**FIGURE 3:**
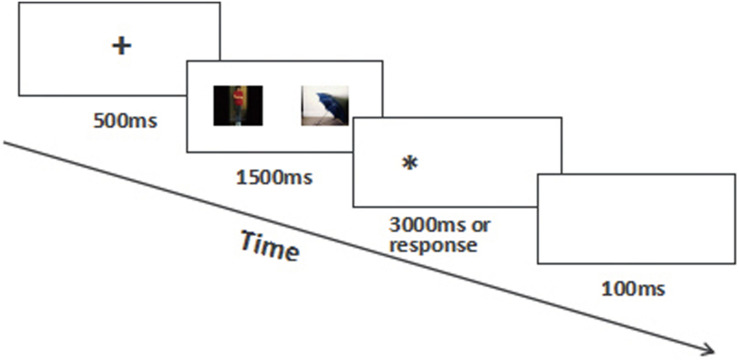
The procedure of Dot-probe task.

Dot-probe task (Macleod et al., 1986) was used in Experiment 2. Each trial began with a + at the center of the screen for 500 ms, followed by a couple of pictures lasting 1500 ms displayed at the left and right of the screen, and then a^∗^ as target presented at the left or right of the screen (one of the locations of the pictures which had been presented before). Finally, the dot target was followed by a blank screen. There were three blocks in the experiment according to the valence of the pictures: neutral–neutral block, neutral–positive block, and neutral–negative block (see Figure 3). To avoid the influence of positive and negative pictures on the response to neutral pictures, the first block was neutral–neutral block, and then flowed the two remaining blocks randomly. The locations of two pictures in each of the blocks were random. There were 70 trials in each block, in which 14 trials (20%) had no target dot in order to avoid participants developing expectancies of the next trial (Song, 2013). There was a short practice block of 10 trials in which the pictures were 4 neutral pictures.

Participants were asked to ignore the pictures and make response to the dot target quickly and accurately by pressing the left or right key corresponding to the position of the target. If there was no dot target after the picture, they just need to do nothing. The target disappeared when the participants pressed the key or appeared more than 3000 ms. The length and width of the ^∗^ was 0.5 cm, the image was 240 × 180 pixels, and the distance between two pictures was 6 cm. The instruments and equipment were the same as Experiment 1.

Like Experiment 1, a week before the lab experiment, participants completed the ER89, SAS, and SDS at first, and the high and low resilient groups were invited to complete the dot-probe task a week later.

### Results

There were no trials with reaction times (RTs) <200 ms or >1000 ms. Accuracy rates of all participants were higher than 96% and were same in two groups, we therefore focused our analysis on RTs. For the RTs analyses, errors were excluded (3.43% of all trials). There was no difference between males and females on the score of ego-resilience [*t*(54) = 1.66, *p* = 0.10]. The detailed RTs of high and low resilient groups under various conditions were shown in Table 4.

**TABLE 4:** The RT of high and low resilient people under various conditions (*M* ± SD).

Position	Neutral–neutral		Neutral–positive		Neutral–negative	
	Left	Right	Neutral	Positive	Neutral	Negative
High	409.2 ± 55.6	401.4 ± 56.7	417.4 ± 68.9	427.9 ± 69.5	414.9 ± 57.2	436.2 ± 67.9
Low	425.9 ± 60.3	412.3 ± 54.4	426.6 ± 59.4	442.9 ± 64.2	440.9 ± 58.5	452.1 ± 54.6

Repeated-measures ANOVA for mean RTs of neutral pictures condition was conducted with Dot’s position (Left, Right) as the within-subject factor, Resilience (High and Low) as the between-subject factor to test whether there was a difference between high and low resilient participants in RT for the left and right position of the target. The results of this analysis showed that the main effect of Dot’s position was significant, *F*(1,54) = 6.91, *p* < 0.05, η^2^ = 0.11, RT of the right dot (*M* = 404.24 ms) was faster than that of the left dot (*M* = 413.59 ms). While the main effect of resilience and the 2-way interaction between Dot’s position and Resilience was not significant, *Fs* < 1 meant that although the participants had distinct responses to the left and right located probe dot, there was no difference between participants with different resiliences in response to the two kinds of dots. We therefore focused on the congruence of the dot and emotional pictures, and there were three conditions, congruence with the neutral (CNa), congruence with the positive (CP), and congruence with the negative (CNe).

Then mean RTs of emotional pictures condition were submitted to a repeated-measures ANOVA with Resilience (High and Low) as between-subject factors and Congruency (CNa, CP, and CNe) as within-subject factors. The results of this analysis showed that the main effect of Congruency was significant, *F*(1, 54) = 31.26, *p* < 0.001, *η*^2^ = 0.37. Follow-up analyses revealed that RT of CNa (*M* = 422.92 ms) was significantly faster than that of CP (*M* = 432.00ms), *F*(1, 54) = 9.49, *p* < 0.05, η^2^ = 0.15, and RT of CP (*M* = 432.00 ms) was significantly faster than that of CNe (*M* = 441.80 ms), *F*(1, 54) = 4.27, *p <* 0.05, η^2^ = 0.07. The main effect of resilience as well as the interaction between Congruency and Resilience were not significant, *Fs* < 1. RT on CNe is longest and on CNa is shortest, but there was no difference in each of the congruency condition between the two groups of resiliences.

In previous studies emotion bias could be Calculated by formula: positive emotion bias = RT_CNa in positive block_ − RT_CP in positive block_; negative emotion bias = RT_CNa in negative block_ − RT_CNe in negative block_ (Bocanegra et al., 2012). Then mean RTs of emotional pictures condition were submitted to a repeated-measures ANOVA with Resilience (High and Low) and Bias (Positive bias and Negative bias) as within-subject factors. The results of this analysis showed that the main effects of Bias and Resilienceas well as the interaction between them was not significant, *Fs* < 1,which meant that resilience had no effect on emotion bias.

### Discussion

The aim of experiment 2 was to explore whether resilience had influences on the experience of emotional information in the input receiving stage of emotional information. The results showed that the selective attention to emotional information of two resilient groups had no significant differences, which may be due to the fact that our participants were screened with a normal range of mental health, while the previous study used individuals with anxiety or depression as their participants (Song, 2013). Due to the results that there was no difference between high and low resilient groups on attention bias, Experiment 3 was further designed to test whether the differences were caused in the stage of emotional disengagement.

## Experiment 3

The results of Experiment 2 showed that there were no significant differences between high and low resilient groups in the stage of receiving emotional information. According to the input–output model, if individuals could rapidly make corresponding emotional responses to the information, they would acquire less emotional experience, with positive materials being rated much lower while negatives being higher. On the contrary, if they spent more time in the disengaging stage, the evaluation of emotional information would be enhanced too. This process called response accommodation (Gross, 1998). To further explore whether the speed of disengaging for information caused the difference in emotional experience between the two groups, the emotional Stroop task was employed in Experiment 3. Studies have shown that the interference effect of emotional Stroop task was caused by the generic slowdown in processing speed and it could reflect the ability to relieve emotional information (Algom et al., 2004; Liu and Wang, 2011).

### Materials and Methods

#### Participants

The participants were selected in the same way as Experiment 1. There were 72 participants recruited in this study (*M* age = 19.11 years, SD = 1.41), 36 (31 male) in the high resilient group, and 36 (32 male) in the low resilient group, the total score of the high psychological resilience group was significantly higher than that in the low group, *t*(71) = 15.81, *p* < 0.001, *d* = 4.26. All participants had normal or corrected-to-normal vision, no history of attention deficit, and all signed the written consent form and were paid for a small monetary reward for the experiment.

#### Materials

Ego-resiliency scale, the Cronbach’s alpha was 0.81. Self-Rating Anxiety Scale, the Cronbach’s alpha was 0.80.Self-rating Depression Scale, the Cronbach’s alpha was 0.81. These pictures being used in emotion Stroop task were the same as Experiment 1.

#### Procedure

**FIGURE 4:**
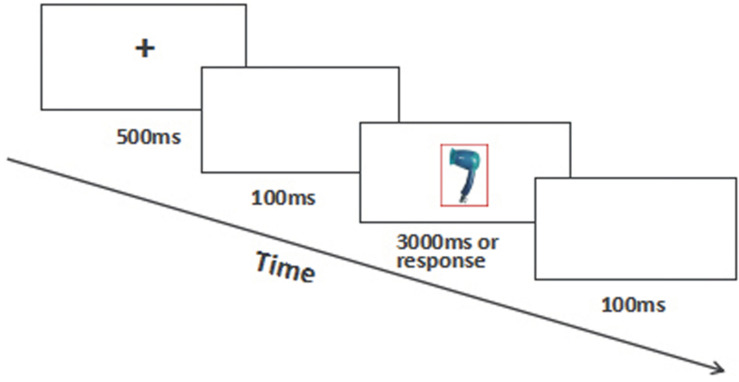
The procedure of emotional Stroop task.

The exchanged form of emotion Stroop task was used to test the ability of disengagement from emotional information (Liu and Wang, 2011). All the pictures were bordered in Experiment 1 with red or green border by using Photoshop CS. Each picture was bordered with two kinds of borders, green and red. The border is 8 pixels (see Figure 4).

Each trial began with a + at the center of the screen for 500 ms, followed by a blank lasting 100 ms, and then a bordered picture was presented and followed by another blank lasting 100 ms again. The same as Experiment 2, there were three blocks in the experiment according to the valence of the pictures: the neutral block, the positive block, and the negative block. To avoid the influence of positive and negative pictures on the response to the neutral pictures, the first block was neutral block, and then flowed the two remaining blocks randomly.

Participants were asked to ignore the pictures and make response to the red and green of the borders by pressing the left or right key. The target disappeared if participants pressed the key or appeared no more than 3000 ms. The instruments, equipments, and experimental procedure were same as Experiment 2.

### Results

One participant was excluded because the accuracy rate was lower than 90%. The RT of trials beyond 3s was excluded either. Accuracy rates of all participants were higher than 96% except one participant and not different in two groups, we therefore focused our analysis on RTs. For the RTs analyses, errors were excluded (7.5%).

Mean RTs of neutral pictures condition were analyzed by repeated-measures ANOVA with Color (Red and Green) as a within-subject factor and Resilience (High and Low) as a between-subject factor to test whether there was a difference between the high and low resilient participants in RT for the red and green colors of the border. The results of this analysis showed that the main effects of Color and Resilience were not significant, *F*(1, 72) = 2.82, *p* = 0.10, η^2^ = 0.04; *F*(1, 72) = 2.64, *p* = 0.11, η^2^ = 0.04, neither does the interaction between Color and Resilience, *F*(1, 72) = 1.25, *p* = 0.27, η^2^ = 0.02, indicating that color had no effect on participants responses, and that there was also no difference between subjects with different resilience in response to the two kinds of pictures. We therefore focused on the emotional type of pictures and resilience.

Mean RTs of three emotional conditions were analyzed by repeated-measures ANOVA with Resilience (High and Low) as a between-subject factor and Emotional type (Nature, Positive, and Negative) as a within-subject factor. The results of this analysis showed that the main effect of Emotional type was significant, *F*(2, 71) = 7.37, *p* = 0.001, η^2^ = 0.093. Follow-up analyses revealed that RT of the neutral pictures (*M* = 477.86 ms) was significantly shorter than that of the positive pictures (*M* = 506.08 ms), *F*(1, 71) = 8.58, *p* = 0.005, η^2^ = 0.105, and the negative pictures (*M* = 522.05), *F*(1, 71) = 13.17, *p* = 0.001, *η*^2^ = 0.16. RT of the positive and negative pictures was not significantly different from each other, *F*(1, 71) = 1.91, *p* = 0.171, η^2^ = 0.03. The main effect of resilience was significant, *F*(1, 72) = 7.89, *p* = 0.006, η^2^ = 0.1, follow-up analyses revealed that the high resilient participants (*M* = 478.45 ms) were significantly faster than the low resilient participants (*M* = 527.77), *F*(1, 73) = 7.89, *p* = 0.006, η^2^ = 0.1. However, the interaction between Emotional type and Resilience was not significant, *Fs <* 1. The results above showed that the response time of the high resilient group was faster than that of the low resilient group, and the negative emotional picture had the longest RT, while the neutral emotional pictures had the shortest RT, and the RT of the positive emotional pictures were in the middle.

**FIGURE 5:**
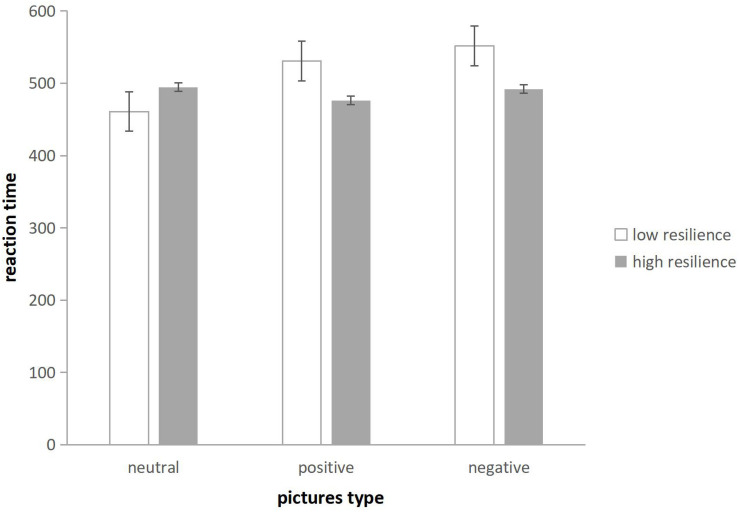
The RT of low and high resilient groups for three types of pictures (Error bars represent the ±1 standard error of the mean).

Independent sample *t* tests indicated that the RT of the high resilient group on positive (*M* = 479.20 ms) and negative (*M* = 495.12 ms) were significantly shorter than that of the low resilient group (*M* = 532.97; *M* = 555.67), *t*(72) = 2.64, *p* = 0.010, Cohen’s *d* = 0.614; *t*(72) = 2.28, *p* = 0.025, Cohen’s *d* = 0.531; see Figure 5. The results showed that the high and low resilient groups were not different in dividing neutral emotional information, but were significantly different in disengaging in positive and negative emotional information.

### Discussion

The results of Experiment 3 showed that the high resilient group are much likely to disengage themselves from both positive and negative emotional information, while the low resilient group tended to immerse in the emotional information, regardless of positive or negative information. This finding was in line with the results of Ong et al. (2006). High resilient people felt something positive compared with the low resilient ones after disasters. This probably because the high resilient participants would quickly come out from the situation, and felt the positive emotions. Therefore, those highly resilient individuals could quickly get out of the negative emotions experience in the disaster, allowing them to have redundant psychological resources to do something else, and receive the positive emotional information.

## General Discussion

There is a prevailing experience for most people that even facing the same thing, different individuals generate various affective experiences. The active role of positive emotion has been supported by a large number of studies (Folkman and Moskowitz, 2004; Koivumaahonkanen et al., 2004; Tugade and Fredrickson, 2004; Dunn and Schweitzer, 2005; Doyle et al., 2006; Fredrickson et al., 2008; Cohn et al., 2009). While previous ideas on negative emotion were inconsistent (Tugade and Fredrickson, 2004; Ong et al., 2006; Bonanno et al., 2007). Nonetheless, it is still unclear what caused these distinct emotional experiences. Some researchers point out that the resilience, as an important factor in coping with stress, adversity, and even daily hassles, may play an indispensable role in the process of emotional experience. In the present study, through conducting the three experiments, we explored the relationships among emotional experience, resilience, and anxiety or depression state, and illuminated that for the same emotional stimulus, the high resilient people felt different from the low resilient ones, the possible reasons might be from the difference at output stage. As we predicted, the high resilient people might tend to make more positive evaluation while the low resilient ones more negative evaluation and hardly disengaging themselves from emotional information.

In the picture rating task, it was found that there was a significant positive correlation between resilience and the emotional experience, and the emotional experience was effectively predicted that the high resilient group rated higher than the low resilient group in both of positive and negative pictures, which means that the experience of the high resilient group on positive pictures are more positive than that of the low resilient group, and the experience of the low resilient group on negative pictures are more negative than that of the high resilient ones. The results of the present study were consistent with the previous research (Anthony and Jenson, 2006), which showed that the high resilient group was easier to perceive positive emotion, while the low resilient group was easier to perceive negative emotion. High resilient individuals could self-generate those positive emotions (Philippe et al., 2018), which allowed them to have a good recovery from stress. Meanwhile, it was also shown that both rating of negative and positive pictures had significantly positive correlation with that of neutral pictures, which might reflect the overall tendency of people to treat their life events or individual idiosyncrasy. In a general way, neutral pictures do not carry emotional information, if one individual rated neutral images higher than the average value, we then could assume that s/he might be an activist, would give more positive evaluation to negative materials as well as positive materials.

In Experiment 1, the symptoms of anxiety and depression of each participant were measured to explore the relationships with resilience. The results showed that there was a negative correlation between resilience and anxiety or depression. This is consistent with the prior researches. For example, Brailovskaia et al. (2017) found that both of resilience and social support had a significant negative association with anxiety and depression, especially in China, there was a stronger association for resilience. It was also pointed out that resilience may potentially moderate the levels of anxiety and depression for patients with anxiety or depression disorders, decreasing the probability of suicide in the prior studies (Min et al., 2014; Gloria and Steinhardt, 2016). Furthermore, it partly mediated negative strategies and acute stress, but fully mediated positive strategies and acute stress (Cai et al., 2017). Indeed, individuals with higher levels of resilience were less likely to suffer from anxiety and depression by linking the results that they evaluate the same thing more positively, and less psychological resources were needed to cope with life-threatening events. The review of Schiele and Domschke (2018) showed that resilient function was associated with positive cognitive emotion regulation strategies, which could decrease the risk of suffering from mental disorders such as depression or anxiety.

There was no difference in the receiving stage of emotional information between high and low resilient groups, which is partly consistent with the previous research (Song, 2013). It was found that in the late stage of attention only the low resilient group still had a negative emotion attention bias. While the attention process was not divided into early and the late stages in the same experiment, we found that the attention bias to both positive and negative emotion pictures of the two groups was not significantly different in Experiment 2, which was conducted to test whether there were differences in the stage of input. Therefore, the difference of emotion rating between high and low resilient groups may not be caused in the input stage of emotional information. The reason why Song’s study (2013) showed an attention bias to negative emotional faces might be because of different kind of materials. Negative faces, such as angry faces, could be processed more quickly than happy faces (Quinlan and Taylor, 2014).

To further explore the reason why the high resilient group felt different from the low resilient group when information input was equal, the emotional Stroop task was used to test the ability of different resilient groups to disengage from emotional information in experiment 3. The results were very interesting. The high and low resilient groups were not different in disengaging from neutral emotional information. However, for the positive and negative emotional information, high resilient group were faster to disengage than the low resilient group did, and the low resilient group was easier to immerse in the emotional information, both positive and negative pictures. Our results of negative emotion were consistent with Song’s (2013) study that high resilient individual could decrease the negative emotional experience and thus improve the mental health level. On the positive trials, high resilient group also disengaged faster from the information than the low resilient group did. This result provides evidence for the uncertainty emotion theory (Pribram, 1967).

The emotion theory of Pribram (1967) suggests that emotion is a disorder of the nervous center. When individuals are confronted with emotional events, whether positive or negative, their balanced state will be interrupted. This is subtly similar to the meta-cognitive model, which emphasizes that dysfunctional metacognitive beliefs about mental state is the basis of commonly emotional disorders (Mathews and Wells, 2004; Wells, 2008, 2013; Thorslund et al., 2020). By maintaining repetitive negative thinking (RNT), these meta-cognitive beliefs will be activated when someone in distress responds to the emotional discomfort. Usually an individual with emotional disorders mistakenly conceived it as a functional plan to deal with reality and its problems (Wells, 2008). Metacognitive therapy, which emerged from the model, also emphasizes that, in order to pull themselves away from painful mental state, the individuals may manage dysfunctional processes through training. So it can be speculated that the high resilient individuals might disengage quickly from emotional information in the daily life, thus can push themselves back to peace and balanced state again, and save psychological resources. Low resilient individuals, however, are easily to be immersed in and maintain repetitive experience of emotions, which could increase the depletion of mental resources and lead to a poor mental health.

As it is well known that negative emotion is not beneficial for individuals’ mental health, immersing in positive emotion is bad as well, which is reflected in the traditional culture of harmony in China. In addition, special edition of the Journal of Personality on “Resilience in Common Life,” Davis et al. (2009) observed that “formost of us, the adversities we encounter do not constitute major disasters but rather are more modest disruptions that are embedded in our everyday lives.” Davydov et al. (2010) also speculated that resilience mechanisms may differ in relation to contextual severity, ranging from resilience against regular everyday hassles like work stress (i.e., mild adversity) to resilience against occasional extensive stress such as bereavement (i.e., strong adversity). Ostensibly positive life events – that are not typically associated with a higher probability of undesirable outcomes – can also be relevant to resilience (Fletcher and Sarkar, 2013). To sum up, it suggests that in the teaching and education, the psychological resilience level of students’ should be fostered consciously, the students’ ability to disengage their emotions away from stressful mental state can be improved to keep their mental health.

The relationships between emotional experience, anxiety and depression tendency, and resilience was revealed in the present study, and it was also shown that the differences of emotional experience between high and low resilient individuals may be due to the differences of the ability to disengage from emotional information. But we just focused on positive, negative, and neutral emotion, and there are several kinds of emotion, actually. It is possible that the different kind of emotion has different relationships with mental health and resilience. At the same time, current research did not verify Ong et al. (2006) findings that high resilient individuals also felt some positive emotions when they felt negative emotions compared with low resilience, which may be because we used a bipolar scale in the emotional rating task, and was not able to assess the presence of both positive and negative effect to the same stimulus (Waugh et al., 2011). Future researches should include more kinds of emotions and use a multidimensional scale that can assess both positive and negative affect simultaneously. In addition, longer scales writing time may also lead to fatigue and affects the reality of data, the SAS and SDS to assess mental health we only used, which may not represent all the mental health comprehensively. Finally, a large number of males was recurited as participants. Although no gender differences in psychological resilience scores, which may still have a small impact on the generalization of the conclusion. More female participants should be recruited in the future research.

## Conclusion

The significance of this study is that it was found the difference of emotional experience among different resilient individuals. Different emotion interventions and training should be designed to enhance mental health according to the differences of individuals’ resilience, which will make the psychological and mental health interventions more targeted and directional. In the future intervention research and teaching practice, it could be considered to enhance the low resilience individuals’ ability to separate themselves from emotional information. By improving the positive emotional experience, decreasing the situation of immersing in negative mood, the risk of anxiety or depression among college students can be reduced.

## Data Availability Statement

The raw data supporting the conclusions of this article will be made available by the authors, without undue reservation.

## Ethics Statement

The studies involving human participants were reviewed and approved by Shaanxi Normal University Ethics Committee. The patients/participants provided their written informed consent to participate in this study.

## Author Contributions

XS and XL conceived and designed the study. XL and FY performed the experiments. LZ provided some advice and comments. XL and FY wrote the manuscript. All authors reviewed, edited, read, and approved the manuscript.

## Conflict of Interest

The authors declare that the research was conducted in the absence of any commercial or financial relationships that could be construed as a potential conflict of interest.
